# ﻿*Schievitermesglobicornis*, a new genus and species of Termitinae (Blattodea, Termitidae) from French Guiana

**DOI:** 10.3897/zookeys.1125.91124

**Published:** 2022-10-20

**Authors:** Yves Roisin

**Affiliations:** 1 Université Libre de Bruxelles, Evolutionary Biology and Ecology, 50 avenue F.D. Roosevelt, 1050 Brussels, Belgium Université Libre de Bruxelles Brussels Belgium

**Keywords:** Isoptera, *Neocapritermes*, Neotropical region, new species, *Planicapritermes*, termite

## Abstract

Asymmetrical snapping mandibles have evolved several times in termites. In the Neotropics, asymmetrical snapping mandibles are found in soldiers of four genera: *Neocapritermes*, *Planicapritermes*, *Cornicapritermes* and *Dihoplotermes*. Here, I describe *Schievitermesglobicornis*, new genus and species, from French Guiana. This genus is characterized by an absence of a frontal prominence and slightly asymmetrical mandibles in the soldier caste. The morphology and anatomy of the worker reveal a wood-based diet, and suggest that *Schievitermes*, *Planicapritermes* and *Neocapritermes* constitute a monophyletic group, which is consistent with mtDNA data.

## ﻿Introduction

Soldiers with snapping mandibles are commonplace in termites, and there is growing evidence that this defensive device evolved several times in the family Termitidae (Inward et al. 2007), as well as once in Kalotermitidae (Scheffrahn et al. 2018). When the soldier presses its mandibles against each other, they accumulate elastic energy, which is converted into kinetic energy as soon as the mandible shafts slip past each other, delivering a very quick and powerful strike (Seid et al. 2008). Although termite mandibles always display some degree of asymmetry, some snapping soldiers can be catalogued as symmetrical because their mandibles both show a similar elongated shape and can deliver a symmetrical blow (Deligne 1971). In asymmetrical snappers, the left mandible is bent outwards in its basal part before straightening apically, whereas the right mandible is almost straight, or slightly bent inwards. When mandibles are pressed against each other, the curved part of the left mandible functions as a spring, concentrating elastic energy in preparation for an asymmetrical blow (Deligne 1971).

Snappers are especially diverse in the Oriental region, but several genera are known from the Neotropics: *Termes* (also present in the Old World tropics), *Cavitermes*, *Palmitermes*, *Crepititermes* and *Inquilinitermes* are symmetrical snappers, whereas *Neocapritermes*, *Planicapritermes*, *Cornicapritermes* and *Dihoplotermes* are asymmetrical ones (Krishna 1968). Asymmetrical snappers appeared separately in the Old World (*Pericapritermes* and related genera), in Madagascar (*Capritermes*) and in the Neotropics, where Inward et al. (2007) suggested that they evolved independently twice, once in *Planicapritermes* and once in *Neocapritermes*. Although more recent studies have established a close relationship between *Planicapritermes* and *Neocapritermes*, forming the sister clade to the symmetrical snapper *Crepititermes* (Bourguignon et al. 2017), it remains that asymmetrical snapping must also have evolved independently in the neotropical *Termes* group (*Dihoplotermes* and probably *Cornicapritermes*). Bourguignon et al.’s (2017) study included a taxon from French Guiana provisionally labelled “G683 *Neocapritermes* sp. H”, which displayed only slightly asymmetrical snapping mandibles and whose mtDNA appeared closer to *Planicapritermes* than to *Neocapritermes*. Reexamination of this sample with more recent collections from French Guiana revealed that this species does not fit into any of the abovementioned genera. I describe it hereunder as *Schievitermesglobicornis*, gen. nov., sp. nov., and discuss its relationships.

## ﻿Material and methods

Dissections were made in alcohol. Guts *in situ* were drawn with a camera lucida. Detached pieces such as mandibles or enteric valves were directly mounted on microscope slides in PVA medium (BioQuip Products Inc.).

Images of entire specimens are multi-layer compilations obtained with a Zeiss Discovery V12 steromicroscope equipped with an AxioCam ICc3 camera and controlled by AxioVision release 4.8.3 software. Images are compilations of series of successive stepwise focused photographs. Images of microscope slide preparations were taken with a Leica DFC450C camera mounted on a Leica DM5500B microscope and operated with Leica Application Suite v.4.12.0 software. Enteric valves and hindgut wall sections were observed under phase-contrast illumination.

Terminology follows that of Sands (1972) for mandible dentition and that of Noirot (1995, 2001) for gut anatomy. Measurements, as described in Roonwal (1969), were taken to the nearest 0.005 mm with a Wild MMS 235 length-measuring set fitted to a Wild M6 stereomicroscope.

## ﻿Taxonomy

### ﻿ Schievitermes gen. nov.

Taxon classificationAnimaliaBlattodeaTermitidae

15F9FD24-BCBA-5FA8-B7C1-72155C54DEE7

https://zoobank.org/9B8C1A8B-047A-4CEC-A182-0288A0593CF4

#### Remark.

This genus is presently monotypic.

#### Type species.

*Schievitermesglobicornis* sp. nov.

#### Description.

***Imago***: only known from a single queen. See species description.

**Figures 1–3.**
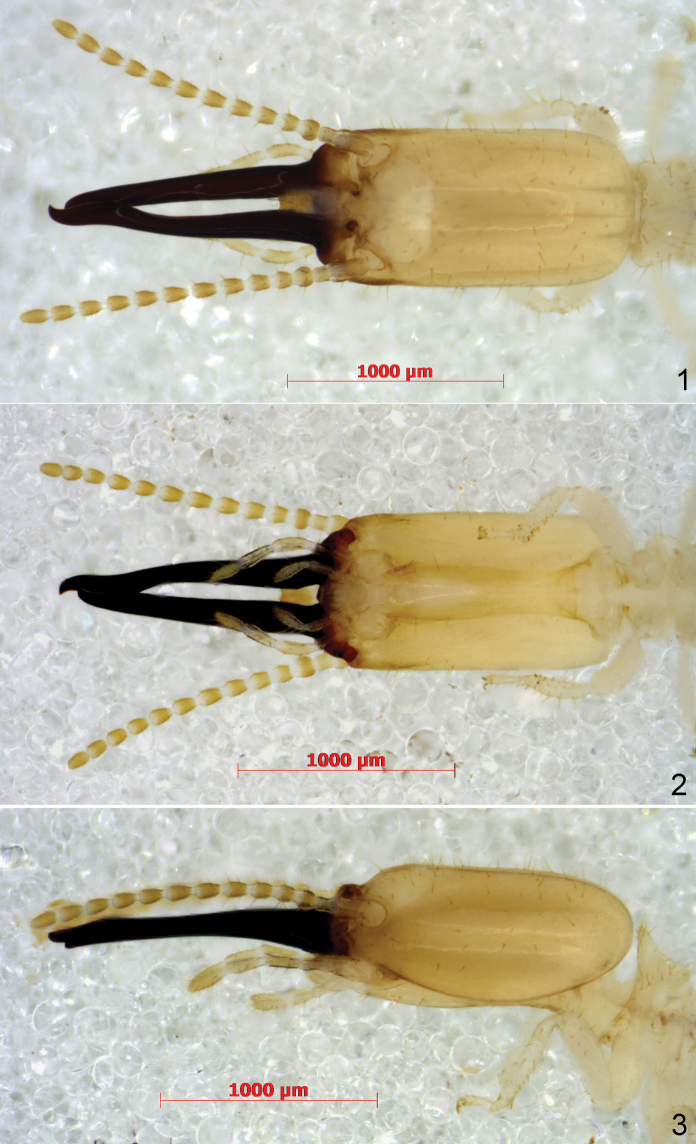
*Schievitermesglobicornis* sp. nov.: soldier head **1** from above **2** from below **3** from left.

**Figures 4–7.**
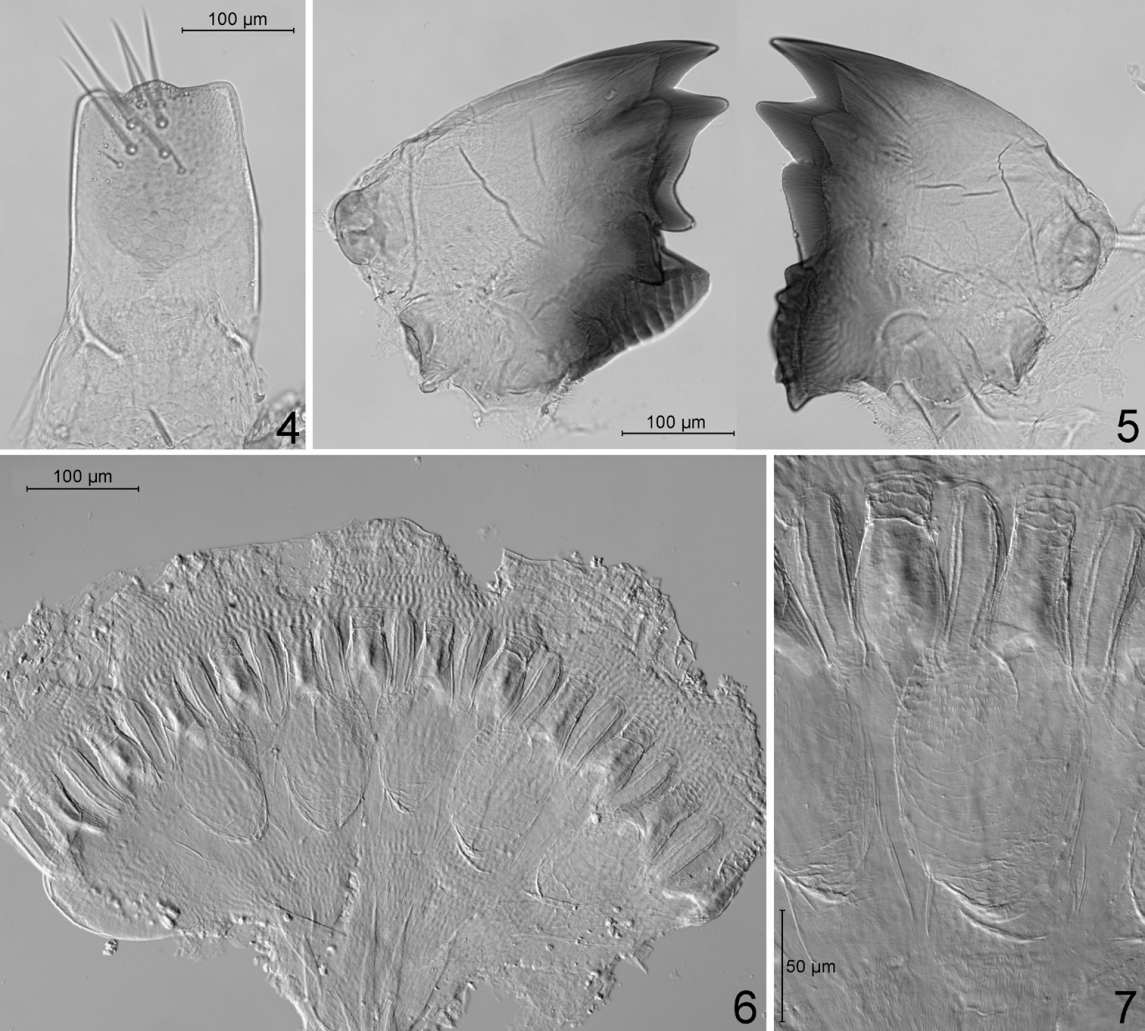
*Schievitermesglobicornis* sp. nov.: **4** labrum of soldier **5** worker mandibles, from above **6** gizzard of worker, complete, spread on slide (phase-contrast illumination) **7** gizzard of worker, detail of pulvillus (phase-contrast illumination).

***Soldier*** (Figs 1–4): Head capsule (Figs 1–3) subquadrangular with rounded corners, about twice as long as broad, bearing numerous (~100) setae. Mandibles approximately as long as head capsule, rather thick, of the snapping type. Right mandible almost straight, only slightly curved inwards. Left mandible slightly but distinctly sinuous: outer margin slightly concave near base, convex in middle, then concave again at level of contact with right mandible, then curved inwards at tip. Tips of both mandibles hooked, turned about 60° inwards. Antennae of 13 articles, apical article reaching beyond left mandible tip; article 3 distinctly globular, as broad as article 1 and broader than all other articles. Labrum (Fig. 4) with nearly parallel sides, anterior margin sinuous, convex in middle, bearing a few long bristles, anterior corners rounded. Frons without projection.

**Figure 8.**
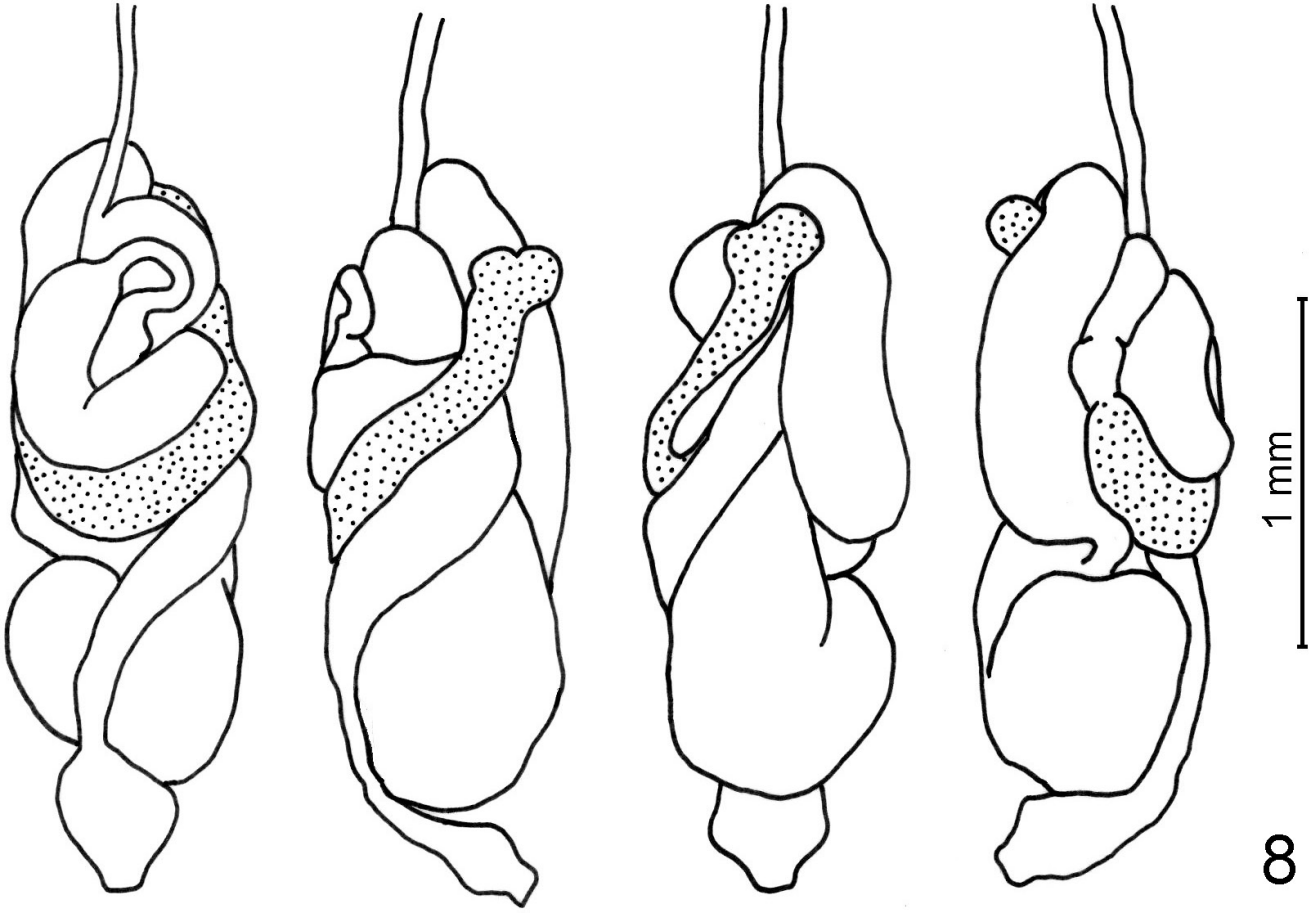
*Schievitermesglobicornis* sp. nov.: camera lucida drawings of worker gut *in situ*. From left to right: viewed from above, right, below, left. Mesenteron stippled.

**Figures 9–14.**
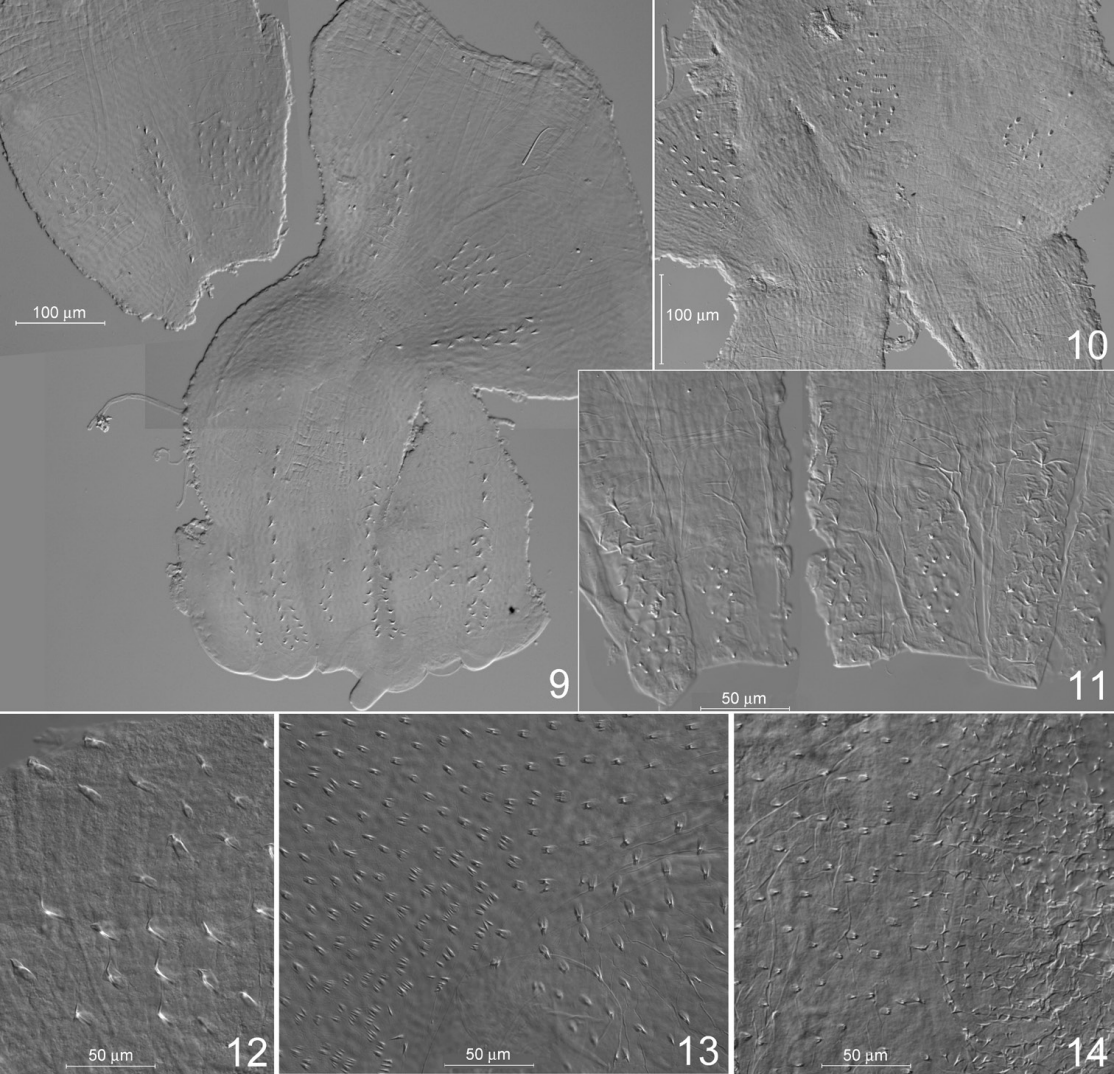
Proctodeal wall ornaments under phase-contrast illumination **9***Schievitermesglobicornis* sp. nov., worker enteric valve, spread on slide **10, 11***Planicapritermesplaniceps* (Emerson, 1925), worker enteric valve, spread on slide; proximal spiny areas and detail of distal spiny ridges, respectively **12, 13***Schievitermesglobicornis* sp. nov., ornamentation of worker paunch wall, in bulbous posterior part and narrower anterior section, respectively **14***Planicapritermesplaniceps*, ornamentation of worker paunch wall.

**Figures 15–17.**
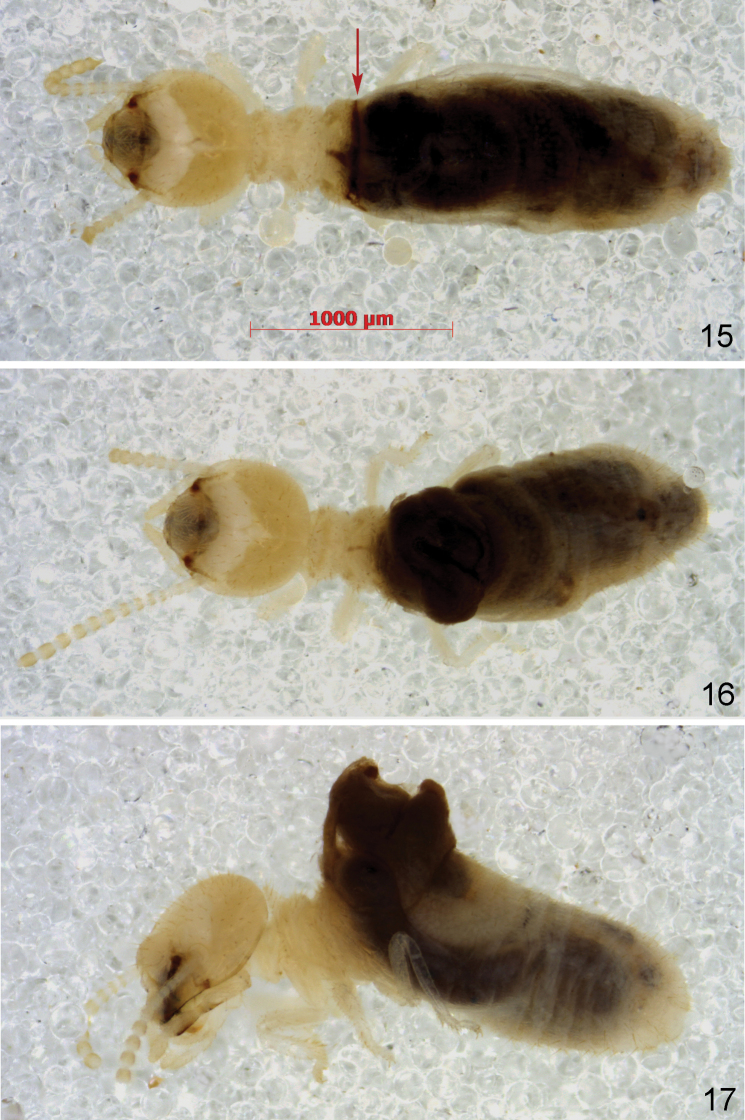
Workers of *Schievitermesglobicornis* sp. nov. fixed at various stages of abdominal dehiscence **15** linear crack (*arrow*) behind rear margin of metanotum **16** gut protruding through crack **17** anterior part of the gut completely extruded.

***Worker*** (Figs 5–9, 12–13, 15–17): Monomorphic. Head capsule whitish, bearing many setae. Mandibles (Fig. 5) of the wood-feeding type. Left mandible: distance between teeth A–M_1+2_ approximately half the distance M_1+2_–M_3_. M_3_ well-marked, premolar tooth (sensu Deligne 1999) blade-like, molar ridges well developed. Right mandible: distance between teeth A–M_1_ short, M_2_ well-marked, molar ridges well developed. Crop moderately developed, gizzard (Figs 6, 7) of the generalized type (Noirot 2001), cuticular armature limited to small pectinated scales on the pulvilli (Fig. 7). Mixed segment long, mesenteric tongue bilobate distally (Fig. 8). Ileum (P1) slightly dilated, narrowing into P2. Enteric valve (P2) funnel-like, conical at end of P1, becoming a narrow tube at junction with P3. Enteric valve armature (Fig. 9) consisting in two rings of spine-bearing areas, the proximal one in the conical section of P2, formed by three ovoid cushions alternating with elongated ones, all bearing small triangular spines; distal ring within the narrow tubular section, formed by six alternating short and long cushions bearing thin, curved spines. Paunch (P3) voluminous, with wall bearing numerous small spines, longer in rounded posterior section near entrance of P2 (Fig. 12), short and often pectinated in anterior section narrowing towards P4 (Fig. 13).

#### Etymology.

From local Brussels dialect *schieve* = not straight, askew, and Latin *termes* = termite. The name refers to the slight grade of asymmetry displayed by soldier mandibles.

#### Diagnosis.

***Soldier***: Among neotropical snappers, the absence of a frontal projection distinguishes *Schievitermes* from *Termes*, *Inquilinitermes*, *Cavitermes*, *Palmitermes*, *Dihoplotermes*, and *Cornicapritermes*. *Planicapritermes* has a characteristic flattened head capsule and strongly asymmetrical mandibles. *Schievitermes* differs from *Crepititermes* by its thicker mandibles with a slight, but distinct asymmetry, and globular third antennal article. *Neocapritermes* species are consistently larger (head width > 1 mm), have more antennal articles (15–16 vs 13) and their mandible asymmetry is always more pronounced (Krishna and Araujo 1968; Constantino 1991; Bandeira and Cancello 1992).

***Worker***: Mandibles are of the wood-feeding type, as in *Neocapritermes* and *Planicapritermes*, with a short space between apical and anterior marginal teeth, and well-developed molar ridges. *Termes* (with the exception of the wood-feeding species, *T.hispaniolae* (Banks, 1918)), *Crepititermes*, *Inquilinitermes*, *Cavitermes*, *Palmitermes*, *Dihoplotermes* and *Cornicapritermes* have mandibles of the soil feeding type, with a broad space between apical and anterior marginal teeth, and reduced molar ridges. The digestive tube of *Schievitermes* is similar to that of *Planicapritermes*, but the bilobed apex of the mesenteric part of the mixed segment is distinctive. *Neocapritermes* also possesses two mesenteric lobes, but the mixed segment is shorter and the mesenteric lobes are larger and more widely separated (Constantino 1998; Almeida-Azevedo et al. 2021). The enteric valve armature of *Schievitermes* is similar to that of *Planicapritermes* (Figs 10, 11), apart from minor differences in the ornamentation of spiny areas. Cuticular differentiations within P3 of *Schievitermes* are intermediate between the long spines and filaments observed in *Neocapritermes* (Noirot 2001) and the tiny spines present in *Planicapritermes* (Noirot 2001; Fig. 14).

Most workers show signs of dehiscence between metanotum and first abdominal tergite, ranging from a short slit-like aperture bordered by brown sclerotic marks (Fig. 15) to a broad opening through which the whole anterior part of the gut protrudes (Figs 16, 17).

### ﻿ Schievitermes globicornis sp. nov.

Taxon classificationAnimaliaBlattodeaTermitidae

77580EFB-B68C-58F6-A4F4-D02B1E9F7293

https://zoobank.org/6D760044-34C4-47E0-8FB9-C3542473928B

#### Material examined.

***Holotype***: soldier. French Guiana, Petit Saut Dam Road, Carbet Maman Lézard, 05.0672°N, 52.9992°W, from nest among tree roots, 20.i.2012 (coll. Y. Roisin – accession G683). ***Paratypes***: soldiers and workers from same colony as holotype. Worker’s mtDNA sequence deposited in GenBank under label *Neocapritermes* sp. H TB-2017 isolate G683, accession KY224444 (Bourguignon et al. 2017). From other colonies than holotype: French Guiana, Laussat (N1 road, PK194), forest on white sands, 05.4698°N, 53.5748°W, small nest in rotten stump, with queen, soldiers and workers, 27.i.2020 (coll. Y. Roisin, N. Fontaine, A. Dumortier – accession G20-12); Mana Road (D8, PK1-2), forest on white sands, 05.5125°N, 53.5504°W, soldiers and workers in dead wood on the ground, 27.x.2021 (coll. Y. Roisin, N. Fontaine, J. Timmermans – accession G21-54). Type material to be deposited in the Royal Belgian Institute for Natural Sciences, Brussels, Belgium.

#### Description.

**Figures 18–20.**
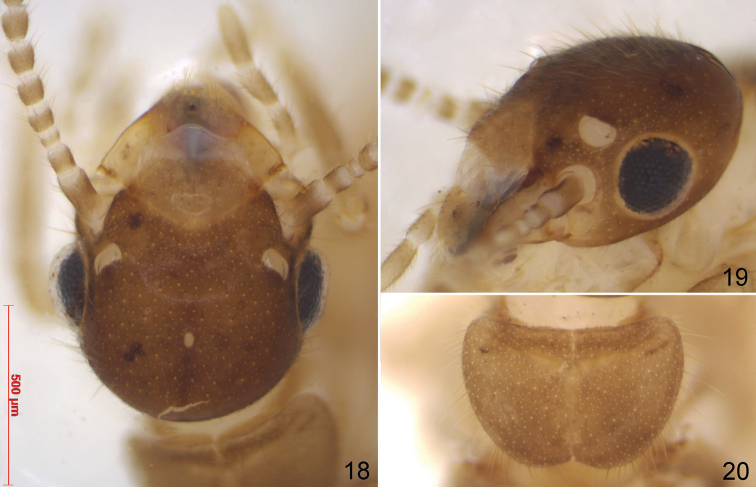
Queen of *Schievitermesglobicornis* sp. nov. **18** head from above **19** oblique view of head **20** pronotum. The three figures are at the same scale.

***Imago*** (Figs 18–20): caste only known from single queen from colony G20-12. This individual was physogastric, with partly depigmented eyes indicating a long underground life. Other body parts were probably of paler pigmentation than in swarming alates as well. Head capsule medium brown, postclypeus, pronotum and other tergites lighter. Antennae broken, 9–11 articles remaining, article 3 narrower and shorter than 2 and 4. Fontanelle hyaline, ovoid, about 45 µm long by 30 µm wide. Head capsule regularly rounded behind. Compound eyes medium-sized, ocelli large, separated from eyes by less than their own width. Pronotum rounded laterally and posteriorly, with a distinct notch behind. Measurements (in mm; numbers between brackets refer to list of measurements proposed by Roonwal 1969): Head length to anterior margin of postclypeus [8]: 0.770; head width, with eyes [17]: 0.810; head width, between eyes [52]: 0.595; eye maximum diameter [48]: 0.260; ocellus maximum diameter [55]: 0.115; ocellus-eye distance [57]: 0.035; pronotum length [65]: 0.425; pronotum width [68]: 0.635; hind tibia length [85]: 0.820.

***Soldier*** (Figs 1–4): Head capsule yellow-brown. Mandibles black, turning brown near base. Antennae yellow-brown. Tibial spurs 3:2:2, anterior spur of fore leg about half the length of the other two.

Measurements of 10 soldiers from three colonies (in mm; numbers between brackets refer to list of measurements proposed by Roonwal 1969): Total head length, including mandibles (held straight forward in measured individuals) [4]: 2.535–2.775; length of head to lateral base of mandibles [5]: 1.320–1.470; head width [17]: 0.700–0.755; length of left mandible [37]: 1.255–1.330; length of right mandible [37]: 1.205–1.295; length of postmentum along median line [61]: 0.800–0.930; maximum width of postmentum [62]: 0.235–0.280; minimum width of postmentum [63]: 0.145–0.175; pronotum width [68]: 0.470–0.510; length of hind tibia [85]: 0.575–0.635.

***Worker*** (Figs 5–9, 12–13, 15–17): Antennae of 13 articles. Tibial spurs 2:2:2. Head width of 20 workers from three colonies: 0.640–0.700 mm.

#### Etymology.

from Latin *globus* = globe, sphere, and *cornu* = horn, antenna; the specific epithet refers to the globular shape of the third antennal article of the soldier.

## ﻿Discussion

Morphologically, *Schievitermes* appears closest to either *Planicapritermes* or *Neocapritermes*. The three genera share asymmetrical snapping mandibles and absence of a frontal projection in the soldier, and worker mandibles revealing wood- or soil-wood interface-feeding habits. *Schievitermes* soldier head and mandible shape appears plesiomorphic with respect to the conspicuously asymmetrical mandibles of *Planicapritermes* and *Neocapritermes*, and the flattened head and body of the former. In the worker, *Schievitermes* is very similar to *Planicapritermes* by its long mixed segment, but the mesenteric lobes at the end of the mixed segment appear intermediate between *Planicapritermes* and *Neocapritermes*. The well-separated lobes of *Neocapritermes* probably represent a derived condition, but the partially bilobed mesenteric tongue of *Schievitermes* might be plesiomorphic, as it appears similar to the condition observed in *Microcerotermes* (Roisin and Pasteels 2000). The enteric valve armature is also very similar in *Schievitermes* and *Planicapritermes*, whereas distal cushions in *Neocapritermes* species are swollen and heavily armed with spines, which probably constitutes a derived trait. The three genera share the presence of numerous spines on the internal wall of P3, although this armature is more complex and comprises longer spines in *Neocapritermes* (Noirot 2001).

Complete mitochondrial DNA sequences have now confirmed the close relationship between *Neocapritermes* and *Planicapritermes* (Bourguignon et al. 2017), as proposed by earlier authors (Krishna 1968; Constantino 1998) but in contrast with the preferred tree of Inward et al. (2007), in which *Planicapritermes* joined a morphologically and zoogeographically improbable lineage including *Orthognathotermes* and *Globitermes*; however, several nodes among the Termitinae were weakly supported, and Inward et al. (2007) did not discard the possible monophyly of *Neocapritermes* + *Planicapritermes*. This new genus *Schievitermes* clearly belongs in the *Neocapritermes* + *Planicapritermes* clade, in which it seems to come closer to *Planicapritermes* in accordance with morphological and anatomical characters.

## Supplementary Material

XML Treatment for Schievitermes

XML Treatment for Schievitermes globicornis
